# Transcriptomic Profiles of *Pectoralis major* Muscles Affected by Spaghetti Meat and Woody Breast in Broiler Chickens

**DOI:** 10.3390/ani14020176

**Published:** 2024-01-05

**Authors:** Sunoh Che, Phuc H. Pham, Shai Barbut, Dorothee Bienzle, Leonardo Susta

**Affiliations:** 1 Department of Pathobiology, Ontario Veterinary College, University of Guelph, Guelph, ON N1G2W1, Canada; ches@uoguelph.ca (S.C.); phpham@uoguelph.ca (P.H.P.);; 2 Department of Food Science, Ontario Agricultural College, University of Guelph, Guelph, ON N1G2W1, Canada; sbarbut@uoguelph.ca

**Keywords:** broiler chicken myopathy, differentially expressed genes, pathogenesis, pathway analysis, RNA sequencing

## Abstract

**Simple Summary:**

We investigated two common muscle conditions in broiler chickens called spaghetti meat and woody breast. These conditions are characterized by changes in the structure and composition of the chicken’s breast muscles. We collected samples from affected and normal muscles and analyzed their genetic profiles. Interestingly, the transcriptomic profiles of spaghetti meat and woody breast samples displayed a high degree of similarity, suggesting that these conditions may have a shared underlying cause. The analysis also revealed that genes related to the extracellular environment and immune response were affected in both conditions. These findings provide valuable insights into the molecular mechanisms of these muscle conditions and may help in developing strategies to mitigate their impact on chicken meat quality.

**Abstract:**

Spaghetti meat (SM) and woody breast (WB) are breast muscle myopathies of broiler chickens, characterized by separation of myofibers and by fibrosis, respectively. This study sought to investigate the transcriptomic profiles of breast muscles affected by SM and WB. Targeted sampling was conducted on a flock to obtain 10 WB, 10 SM, and 10 Normal *Pectoralis major* muscle samples from 37-day-old male chickens. Total RNA was extracted, cDNA was used for pair-end sequencing, and differentially expressed genes (DEGs) were determined by a false discovery rate of <0.1 and a >1.5-fold change. Principal component and heatmap cluster analyses showed that the SM and WB samples clustered together. No DEGs were observed between SM and WB fillets, while a total of 4018 and 2323 DEGs were found when comparing SM and WB, respectively, against Normal samples. In both the SM and WB samples, Gene Ontology terms associated with extracellular environment and immune response were enriched. The KEGG analysis showed enrichment of cytokine–cytokine receptor interaction and extracellular matrix–receptor interaction pathways in both myopathies. Although SM and WB are macroscopically different, the similar transcriptomic profiles suggest that these conditions may share a common pathogenesis. This is the first study to compare the transcriptomes of SM and WB, and it showed that, while both myopathies had profiles different from the normal breast muscle, SM and WB were similar, with comparable enriched metabolic pathways and processes despite presenting markedly different macroscopic features.

## 1. Introduction

Spaghetti meat (SM) and woody breast (WB) are emerging breast myopathies observed today in fast-growing commercial broiler chickens. SM is characterized by the unraveling and splitting of muscle fibers [1], whereas WB is characterized by abnormal firmness due to increased fibrous tissue, pale color, and occasional petechial hemorrhages with variable amounts of exudate on the epimysial surface [2]. Fillets may be affected by SM and WB individually or together in the same fillet [3]. The myopathies do not constitute a public health concern since neither biological nor chemical hazards have been associated with them [4,5]; however, alterations in severely affected fillets can negatively influence meat processability (e.g., higher drip and cooking losses) and consumer acceptance, leading to downgrading or rejection of affected meat, and ultimately causing significant economic losses [4,6,7]. The myopathies have also been associated with decreased nutritional values as they can have lower protein content and higher fat levels compared to normal fillets [4,8].

Although SM- and WB-affected fillets differ macroscopically, they share common histological changes consisting of polyphasic myofiber degeneration and necrosis (hyalinized fibers, loss of cross-striations, and myofiber fragmentation), perivascular accumulation of inflammatory cells (mainly lymphocytes and macrophages with a few heterophils), and endomysial accretion of fibrous tissue and adipocytes [2,9,10].

The pathogenesis of these myopathies is incompletely understood [8,11,12]. Since they occur more commonly in fast-growing heavy strains of broiler chickens [8,13,14], a genetic predisposition of SM and WB has been suggested [15,16,17]. However, since the growth rate is also dependent on management practices, environmental (non-genetic) factors likely also contribute to the development of both SM and WB [3]. While the effects of environmental (non-genetic) factors on SM remain to be determined, several studies have suggested that such factors are likely to contribute to WB [16,17,18].

Transcriptomic studies identified multiple pathways involved in the development of WB, including dysregulation of energy metabolism, immune and inflammatory responses, vascular disease, and remodeling of extracellular matrix [19,20,21]. In one study, desmin and vimentin (key proteins of myocyte architecture) were shown to be upregulated in SM-affected fillets using quantitative PCR [22]. However, the breadth of metabolic and inflammatory pathways contributing to the development of breast muscle myopathies remains to be fully elucidated [8], especially for SM, which is a relatively recent myopathy to be recognized [23].

This study aimed to determine and compare the transcriptomic profiles of breast muscles affected by SM and WB. We hypothesized that the myopathies have distinct transcriptomic signatures relative to each other and relative to normal breast fillets. A more detailed understanding of the pathogenesis of these lesions could inform future mitigating strategies.

## 2. Materials and Methods

### 2.1. Sample Procurement and Classification

All muscle samples were collected during one visit at a processing plant in Ontario in April 2021 and derived from the same broiler flock of 37-day-old male chickens (Ross 708 strain). The flock had a 2.23 kg target live weight, which is in line with the market demand for fresh whole chicken breast sold in Ontario. Fillets were directly sampled from a conveyor belt in the deboning area of the processing plant (line speed of 200–250 birds/min) until 10 samples of each Normal, SM and WB groups were obtained based on the inclusion criteria specified below (targeted sampling). The decision to collect 10 samples per group was based on a previous study [20]. No live animals were directly employed for this study. Samples were classified as affected by SM if myofiber separation was noted upon visual inspection and had no evidence of WB, as evaluated by palpation and visual inspection (Figure S1a). Woody breast was initially graded as absent, moderate, or severe through both visual inspection (bulging) and palpation (increased levels of firmness), as previously described [10] (Figure S1b). To improve the reproducibility of scoring, and avoid intermediate (moderate) phenotypes, only fillets exhibiting severe hardening throughout the tissue were identified as WB and included in the study. Further, to be classified as WB, fillets must not have shown any macroscopic evidence of SM. Fillets without macroscopic abnormalities were classified as Normal (Figure S1c). White striping (WS), another form of broiler breast myopathy, was not included in the macroscopic classification or data stratification since most samples have some degree of WS, consistent with our previous findings on a similar cohort [3]. Fillets (*Pectoralis major*) were classified by an experienced team member (S.C.) with the help of plant workers and obtained from the deboning area of the plant. Ten samples from each group (SM, WB, and Normal) were used.

All samples were procured within 3 h post-slaughter. Using sterile scalpel blades, 1–2 g pieces of tissue were collected from the most severely affected area of each WB- or SM-affected fillet; a similar-size sample was collected from the cranial and superficial aspect of the Normal fillets. The excised tissue was immediately placed in a tube containing 2 mL of 0.5 M EDTA, 25 mM sodium citrate, 70% (*w*/*v*) ammonium sulfate at a pH of 5.2, and frozen at −80 °C. Histology was not performed due to lack of clearly defined histological criteria for differentiation of these myopathies and the high degree of overlapping microscopic changes often observed in affected tissues [9,10], which might have prevented appropriate sample stratification. Macroscopic evaluation was adopted as it remains the most common and relevant classification method employed in the field.

### 2.2. RNA Isolation, Quantification, and Quality Assessment

Total RNA was extracted from samples with E.Z.N.A^®^ Total RNA Kit II (Omega Bio-Tek, Norcross, GA, USA) according to the manufacturer’s protocol. The RNA was quantified using a spectrophotometer (Nanodrop, Thermo Fisher Scientific, Madison, WI, USA), and RNA integrity was assessed with a 2100 Bioanalyzer (Agilent Technologies, Santa Clara, CA, USA) at the central genomics facility of the University of Guelph. All samples had an RNA integrity number (RIN) greater than 7, which was considered suitable for cDNA library preparation.

### 2.3. Library Preparation and Sequencing

At Genome Quebec (Montreal, QC, Canada), the quality of RNA samples, library preparation, and sequencing were assessed using similar methods to those previously described [24]. All 30 samples passed the initial RNA quality control, and cDNA libraries were generated utilizing 250 ng of RNA from each sample. Subsequently, the Next Poly(A) Magnetic Isolation Module (New England BioLabs, Whitby, ON, Canada) was employed to enrich the mRNA, followed by cDNA synthesis through the RNA First Strand Synthesis and Next Ultra Directional RNA Second Strand Synthesis Modules (New England BioLabs). The Next Ultra II DNA Library Prep Kit for Illumina (New England BioLabs) was implemented for DNA fragmentation, adaptor ligation, library amplification, and library analysis. The library was then quantified using the KAPA Library Quantification Kits (Kapa Biosystems, Indianapolis, IN, USA). The average fragment size was determined using the LabChip GX (PerkinElmer, Waltham, MA, USA). The library was normalized and denatured in 0.05N NaOH, and the pH neutralized with hybridization (HT1) buffer (Illumina, San Diego, CA, USA). Paired-end sequencing was performed using the Illumina NovaSeq S4 lane incorporating a 1% phiX library concentration as control. The Real-Time Analysis (RTA) software (v3.4.4; Illumina) was used for base calling, and the *Bcl2fastq2* conversion software (v2.20; Illumina) was employed to demultiplex samples and generate FASTQ reads.

### 2.4. Sequence Quality Assessment, Alignment, and DEG Analysis

Adaptor sequences and bases with a low-quality score (Phred score < 30) were trimmed from reads using the open-source software Trimmomatic v.0.39 (http://www.usadellab.org/cms/index.php?page=trimmomatic assessed on 1 April 2022) [25]. The resulting reads were aligned to the chicken genome (Gallus_gallus_GRCg6a genome) using Spliced Transcripts Alignment to a Reference v2.7.10a (STAR; https://code.google.com/archive/p/rna-star/ assessed on 15 April 2022) [26]. Read counts were obtained using HTSeq v2.0 (http://www-huber.embl.de/HTSeq assessed on 20 April 2022) [27]. The R package limma in Bioconductor v3.15 (https://bioconductor.org/packages/release/bioc/html/limma.html assessed on 30 April 2022) [28] was employed to identify differences in expression levels between groups and to visualize the gene expression data using PCA plot and heatmaps. Evaluation of DEGs was conducted by comparing SM vs. Normal, WB vs. Normal, and SM vs. WB. The significance threshold for calling DEGs was set at a false discovery rate (FDR) < 0.1 according to the Benjamini–Hochberg method, applying a minimum fold change of 1.5. These parameters were chosen due to the exploratory nature of the study, and to increase our sensitivity to detect possible transcriptomic associations between genetically highly homogeneous samples. Lastly, limma was also used to evaluate the direction of change for each gene (i.e., up- or downregulated).

### 2.5. Pathway Enrichment Analysis

In order to evaluate DEG within the pathway framework, two functional enrichment analyses were performed with gProfileR v1 (http://biit.cs.ut.ee/gprofiler/ assessed on 10 May 2022) [29]: Gene Ontology (GO) and Kyoto Encyclopedia of Genes and Genomes (KEGG). Enrichment *p*-values were derived from a hypergeometric test using a set of known chicken genes as background. The genome assembly of Gallus_gallus.GRCg6a.Ensembl98.gtf was used as the reference genome. To reduce false positive findings, the default Set Counts and Sizes method (g:SCS method) was applied for multiple testing corrections for *p*-values obtained from the GO and KEGG pathway enrichment analyses. An adjusted *p*-value threshold of 0.05 was employed. The top 20 enriched terms were used for the GO enrichment count plot.

## 3. Results

### 3.1. Sequence Quality Assessment and Alignment

A summary of pre-processing procedures and quality assessment is provided in Table S1. The number of raw reads per sample ranged from 161,705,438 to 246,025,724. After removing adaptor sequences and low-quality reads (Phred score < 30), between 85.31% and 91.36% of the original raw reads were mapped against the chicken reference genome. The number of mapped reads per sample ranged from 140,385,186 to 224,652,618, and, for each sample, the reads were mapped to a range of 10,943 to 11,766 genes.

### 3.2. Principal Component Analysis (PCA)

**Figure 1:**
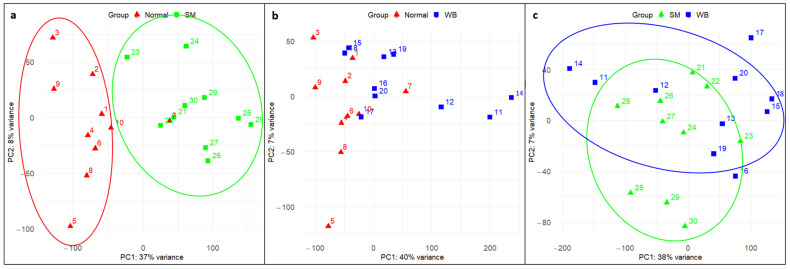
Principal component analysis (PCA) plot showing the clustering pattern of Normal, spaghetti meat (SM), and woody breast (WB) samples. The horizontal axis shows the first dimension of the principal component analysis (PC1), and the vertical axis shows the second dimension of the principal component analysis (PC2). (**a**) Normal vs. SM. Along the PC1 axis, Normal samples cluster together (red ellipse) and are distinct from SM, which cluster together (green ellipse). Along the PC2 axis, samples do not cluster by myopathy group. PC1 and PC2 capture 37% and 8%, respectively, of the variations between the groups. (**b**) Normal vs. WB. Normal samples cluster together (red ellipse) along PC1, while WB samples show some dispersion along the PC1 axis (blue ellipse). PC1 and PC2 capture 40% and 7%, respectively, of the variations between the groups. (**c**) SM vs. WB. Along the PC1 axis, SM samples appear to cluster more closely (green ellipse) compared to WB samples, which are dispersed (blue ellipse). PC1 and PC2 capture 38% and 7% of the variations between the groups, respectively.

PCA plots were drawn for each comparison, showing that PC1 and PC2 dimensions explained 37–40% and 7–8% of the total variance, respectively (Figure 1a–c). Along the PC1 axis, the majority of the Normal samples clustered separately from the SM samples, except for sample number 7 (a possible outlier, Figure 1a). The Normal and WB samples showed separation along the PC1 axis, although overlap was present. While the WB samples appeared to cluster along the PC2 axis, this feature represents a smaller proportion (7%) of the total variance (Figure 1b). The SM samples were clustered along the PC1 axis, while the WB samples were dispersed along the PC1 axis (Figure 1c). Overall, the PCA analysis showed that the most obvious sample separation occurred between Normal and SM fillets, while the most overlap was observed between the SM and WB samples.

### 3.3. Analysis of Differentially Expressed Genes (DEGs)

**Figure 2:**
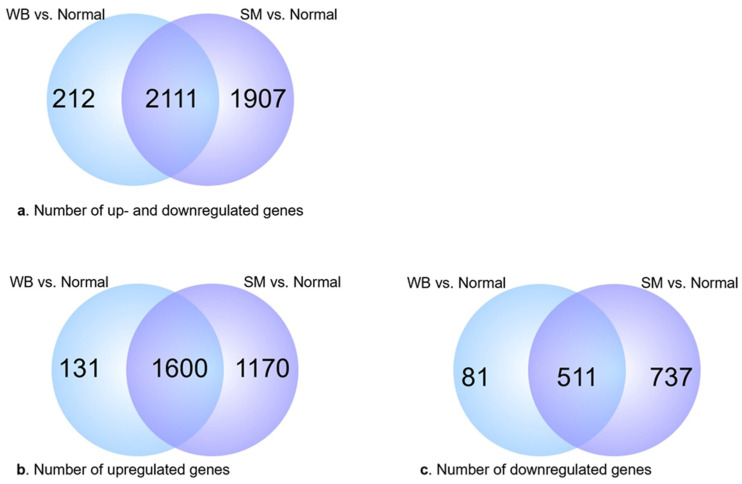
Venn diagram of genes differentially expressed in woody breast (WB) vs. Normal, and spaghetti meat (SM) vs. Normal. Overlapping areas indicate common differentially expressed genes. (**a**) Total number of up- or downregulated genes. (**b**) Number of upregulated genes. (**c**) Number of downregulated genes. Circles are not drawn to scale.

Analysis of differentially expressed genes (DEGs) was performed for three sets of comparisons: (1) SM vs. Normal, (2) WB vs. Normal, and (3) SM vs. WB. The latter comparison (SM vs. WB) did not yield significant DEGs and was excluded from further analysis; therefore, all data reported hereon are in comparison to the Normal group. The SM samples yielded a total of 4018 DEGs. Of these, 2770 (68.9%) were upregulated and 1248 (31.1%) were downregulated. In the WB group, there were a total of 2323 DEGs; of these, 1731 (74.5%) were upregulated and 592 (25.5%) were downregulated. Between the SM and WB groups, there were 2111 common DEGs (Figure 2). Unknown genes (i.e., genes not identified in the annotated genome) accounted for 25.2% in both comparisons: 1011/4018 in SM and 585/2323 in WB.

**Figure 3:**
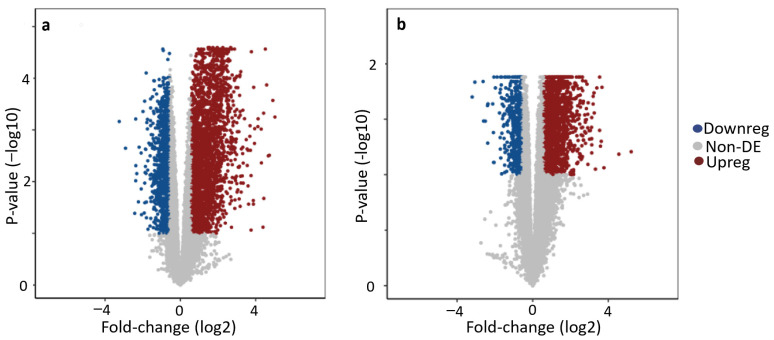
Volcano plots of differentially expressed genes. The volcano plot showing fold changes and statistical significance of genes that are differentially expressed between spaghetti meat (SM) and Normal (**a**), and between woody breast (WB) and Normal (**b**). The most highly upregulated genes are on the right, the most downregulated genes are on the left, and the most statistically significant genes are towards the top. Blue dots represent significantly downregulated genes (fold change < 1.5, false discovery rate < 0.1), and red dots represent significantly upregulated genes (fold change > 1.5, false discovery rate < 0.1). Gray dots represent genes that were not significantly differentially expressed.

Volcano plots (Figure 3) showed that the SM and WB samples had a similar range of fold changes in gene expression, spanning from −3.24 to 5.02, and −3.20 to 5.21, respectively. The majority of the DEGs (95%) ranged between a −0.9 and 2.1 log2 fold change in SM, and −0.8 and 1.6 log2 fold change in WB. The range of significance for DEGs was higher for SM (−log10 *p*-value, 3.0 × 10^−6^–4.59) compared to WB (−log10 *p*-value, 1.2 × 10^−4^–1.88).

The top 20 upregulated and 20 downregulated known genes (i.e., identified in the annotated genome) in the SM and WB samples are presented in Table 1 and Table 2, respectively. Of these 80 genes, 14 were upregulated and 11 downregulated in both SM and WB (with the same directionality, total = 25), and all the genes that were differentially regulated in both groups had the same direction of expression (Table 3).

**Figure 4:**
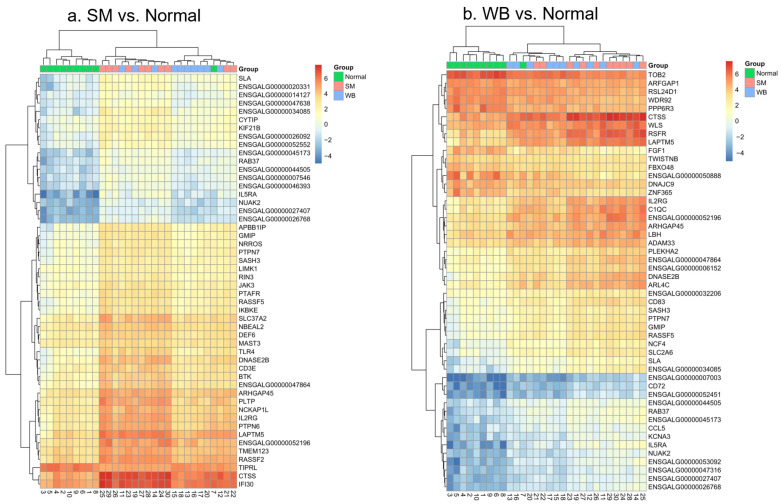
Clustered log counts per million (logCPM)-based heatmaps for the top 50 differentially expressed genes in (**a**) spaghetti meat (SM) and (**b**) woody breast (WB), as compared to Normal. WB and SM outgroups were included in figures a and b, respectively, to provide context. The gene name is presented in each row, and the samples are color-coded and identified by column.

Heatmaps were produced using the log counts per million (logCPM) values to visualize the absolute expression of the top 50 DEGs when comparing SM and WB against Normal samples (Figure 4a and Figure 4b, respectively). In each pairwise comparison, the other myopathy category was added to the plots as an outgroup to provide additional context about myopathy clustering. In both analyses, the Normal samples clustered separately from the SM and WB samples, while SM and WB did not form distinct groupings (Figure 4a,b), consistent with a lack of DEGs between SM and WB.

Samples identified as either SM or WB had upregulated expression of genes linked to various processes, including proteins degradation (e.g., cathepsin S (*CTSS*), gamma-interferon-inducible lysosomal thiol reductase (*IFI30*), and lysosomal protein transmembrane 5 (*LAPTM5*); RNA breakdown (ribonuclease superfamily-related (*RSFR*)); signaling molecule, which plays a role in the development and maintenance of tissues (Wnt ligand secretion mediator (*WLS*)); immune system signaling (interleukin 2 receptor subunit gamma (*IL2RG*)); and complement system and immune defense (complement C1qC chain (*C1QC*)). Normal samples showed decreased expression of a block of genes involved in the immune response and cell metabolism, such as B-cell differentiation antigen CD72 molecule (*CD72*), C–C motif chemokine ligand 5 (*CCL5*), interleukin 5 receptor subunit alpha (*IL5A*), potassium voltage-gated channel subfamily alpha (*KGNA3*), and SNF1/AMP kinase-related kinase (*NUAK2*).

### 3.4. Collagen-Related DEGs

To gain additional insights into the extracellular environment of myopathy samples, we evaluated the expression of collagen genes in our cohort since different types of collagen have been previously shown to be increased in WB [30,31]. A total of 13 collagen genes, spanning from collagen type I alpha 1 chain (*COL1A1*) to collagen type XIII alpha 1 chain (*COL13A1*), were upregulated in both SM and WB (Table 4). Of these, the log2 fold change was highest in collagen type XII alpha 1 chain (*COL12A1*) and collagen type VIII alpha 2 chain (*COL8A2*) in both SM and WB. Two additional collagen genes (collagen type XIV alpha 1 chain [*COL14A1*] and collagen type V alpha 1 chain [*COL5A1*]) were upregulated in SM samples only. Overall, upregulation of these genes spanned between 0.83 and 2.12 log2 fold changes, and no collagen genes were upregulated exclusively in the WB samples. In the comparisons between SM and Normal samples, the adjusted *p*-values ranged from 7.15 × 10^−4^ (for *COL6A3*) to 2.58 × 10^−2^ (for *COL8A2*), while, in the comparisons between WB and Normal samples, they ranged between 2.46 × 10^−2^ (for *COL9A2*) and 6.14 × 10^−2^ (for *COL3A1*) (Table 4).

### 3.5. Gene Ontology (GO) Enrichment Analysis

**Figure 5:**
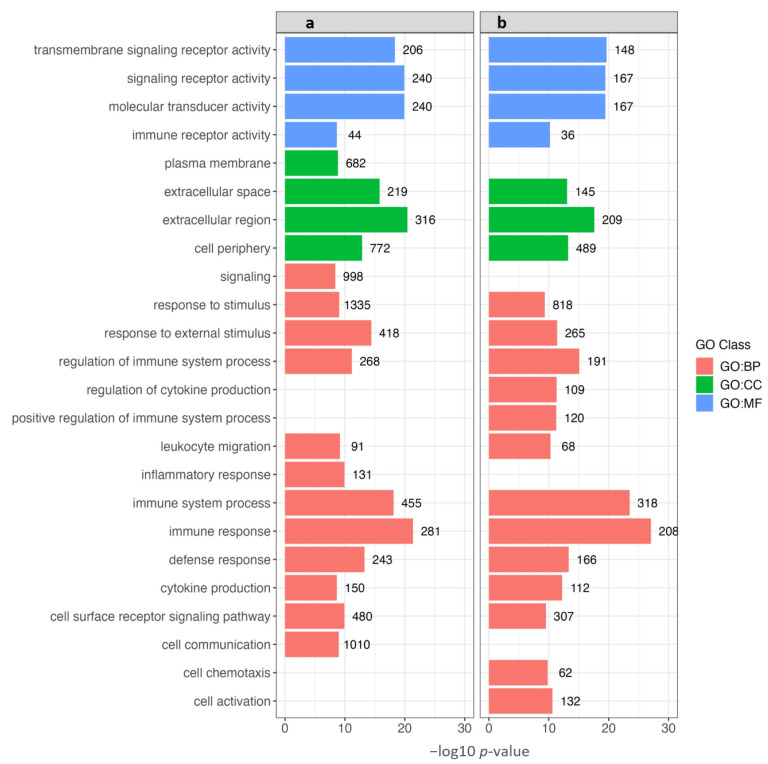
Gene Ontology (GO) enrichment analysis of differentially expressed genes in (**a**) spaghetti meat (SM) and (**b**) woody breast (WB) compared to Normal. The top 20 significantly enriched GO terms are shown for each comparison. Red color represents the *Biological Process* (BP) category; green represents the *Cellular Component* (CC) category; and blue represents the *Molecular Function* (MF) category. The bar indicates the level of significance (−log10 *p*-value), and the numbers adjacent to bars indicate number of genes enriched in that term.

The functional significance of the DEGs identified in the SM and WB samples (as compared to the Normal group) was investigated using GO enrichment analysis. The number of genes for each enriched GO term ranged between 36 and 1335. For each comparison, the top 20 enriched GO terms were plotted. Considering SM, twelve of these belonged to the *Biological Process*, four to the *Cellular Component*, and four to the *Molecular Function* category (Figure 5a). For WB, thirteen of the top enriched terms belonged to the *Biological Process*, three to the *Cellular Component*, and four to the *Molecular Function* category (Figure 5b). For both SM and WB, the *Biological Processes* category was dominated by the enrichment of GO terms associated with immune functions, including response to stimulus, response to external stimulus, regulation of immune system process, regulation of cytokine production, positive regulation of immune system process, leukocyte migration, inflammatory response, immune system process, immune response, and defense response (Figure 5a,b). For both sets of samples, immune system process and immune response had the highest level of significance (− log10 *p*-value > 24) among all the enriched GO terms. In both myopathies, the *Cellular Component* categories included GO terms associated with the pericellular space, such as the extracellular region, extracellular space, and cell periphery. The *Molecular Function* category showed the most significant enrichment of GO terms associated with cell signaling and signal transduction.

### 3.6. Kyoto Encyclopedia of Genes and Genomes (KEGG) Pathway Analysis

**Figure 6:**
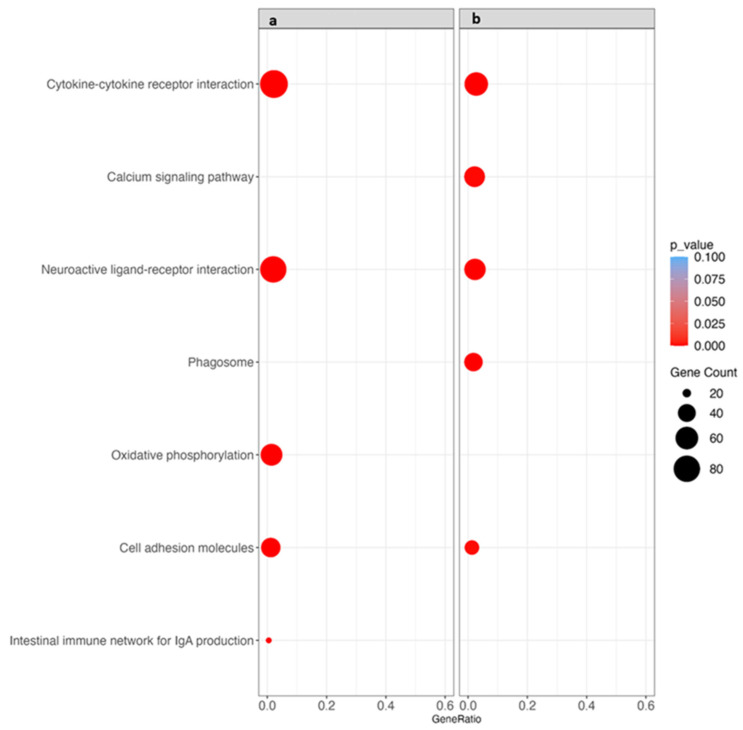
Kyoto Encyclopedia of Genes and Genomes (KEGG) pathway enrichment analysis. (**a**) Spaghetti meat (SM) vs. Normal; (**b**) woody breast (WB) vs. Normal. The size of the circle represents the numbers of differentially expressed genes per KEGG pathway, and the color indicates the adjusted *p*-value.

The KEGG pathway enrichment analysis showed five significantly enriched pathways in both the SM and WB samples (as compared to Normal). Overall, the enriched pathways included between 19 and 89 genes. Common to both SM and WB, three pathways were enriched: cytokine–cytokine receptor interaction, neuroactive ligand–receptor interaction, and cell adhesion molecules. Oxidative phosphorylation and intestinal immune network for IgA production pathways were enriched in the SM samples only. Calcium signaling pathway and phagosome pathways were enriched in the WB samples only (Figure 6). Table S2 details the list of up- and downregulated genes in each enriched KEGG pathway.

## 4. Discussion

Next-generation RNA sequencing (RNA-seq) is a method to quantify the expression of all genes in an agnostic fashion [32,33]. In this study, we used RNA-seq to investigate the transcriptomic profiles of SM and WB samples to identify the genes and pathways involved in their pathogenesis. We hypothesized that these myopathies have distinct transcriptomic signatures relative to each other and to normal breast samples. Despite using an FDR threshold of 10% to increase sensitivity to detect more DEGs between groups, we found that SM and WB did not have different transcriptomic profiles.

Evaluation of differences between DEGs, as well as PCA and heatmap cluster analyses, showed minimal to no differences between the transcriptomic profiles of SM and WB. In contrast, Normal samples could be separated from both SM and WB, albeit this difference was more stark regarding SM compared to the WB samples. Overall, the WB samples were more dispersed compared to the SM samples, which could explain the lack of DEGs detected between SM and WB. This also suggests that the WB cohort in our study might have included varying degrees of severity, likely because of low reproducibility of the score/assessment for WB. On the other hand, less variance in the SM group indicated a more homogenous set of samples, possibly due to the obvious selection threshold imposed by the myofiber unravelling.

The minimal differences observed in the transcriptomic profiles of SM and WB is a finding consistent with the numerous histological characteristics shared between these myopathies [10] (albeit SM appears to have specific microscopic changes, such as collagen rarefaction [9]). Overall, a lack of substantial DEGs suggests that SM and WB may share common genetic characteristics in spite of macroscopically distinct phenotypes [4,10]. It is worth noting that WB sample #7 deviated from the expected pattern and was considered an outlier. However, this was retained in the analysis to account for its potential impact as excluding a biological outlier may lead to an underestimation of variance.

The fact that numerous genes were differentially upregulated, yielding several enriched pathways, suggests that SM and WB can induce complex alterations in muscular homeostasis. These changes are compatible with the previously reported microscopic lesions observed in these fillets, such as myofiber degeneration, acute and chronic inflammation, and repair (mainly in the form of increased fibrous and adipose tissues in the endomysial space) [2,10,34,35]. The following segment of the discussion focuses on the main metabolic pathways enriched by GO and KEGG analyses.

### 4.1. Inflammation and Cytokine Signaling

In both SM and WB, there was enrichment of GO terms associated with immune response, as well as the *cytokine–cytokine receptor interaction* KEGG pathway, suggesting activation of inflammation and immune processes. This result is in agreement with our previous study, where we showed that both SM and WB (same source as samples in this study) had myositis, predominately characterized by lymphocytic cuffs around venules in the endomysial space [10]. Other studies also identified inflammation as a common change in broiler breast myopathies, particularly WB [2,9,10,11,34].

In both SM and WB, numerous cytokines and their receptors were upregulated, including IL1, IL2, IL5, IL11, IL12, IL13, IL17, IL18, and IL20–22. These molecules are generally pro-inflammatory; however, how their net effect influences the phenotype of SM and WB remains to be elucidated. Numerous genes coding for C–C motif chemokines and related receptors were also upregulated in our samples, including *CCL4*–*8*. The function of these molecules is to attract inflammatory cells to the site of inflammation, which is consistent with observing inflammatory cells in the endomysium in SM and WB. Similarly, it was demonstrated that *CCL5* was upregulated in the muscle tissue of modern fast-growing broilers, whereas it was downregulated in a traditional broiler line [36]. Additional upregulated genes in the *cytokine–cytokine receptor interaction* KEGG pathway include transforming growth factor B (*TGFB*) and platelet derived growth factor receptor (*PDGFRA* and *B*). TGF has a pro-fibrotic effect, possibly through the exhaustion of satellite cells [37]. While *PDGFRA* and *B* were upregulated in SM samples only, upregulation of these genes may indicate activation and/or proliferation of fibro-adipogenic precursors, consistent with the accretion of fibrofatty tissue in the endomysium of SM [38].

Three genes associated with inflammation were among the top twenty upregulated genes in both SM and WB: pentraxin 3 (*PTX3*), macrophage receptor with collagenous structure (*MARCO*), and P53 apoptosis effector related to PMP-22 (*PERP1*). PTX3 is a mediator of acute inflammation and innate immunity; in chickens, it is upregulated in tissues upon stimulation with lipopolysaccharide and experimental infection with *E. coli* [39,40]. MARCO is a scavenger receptor expressed on the macrophage surface and mediates opsonin-independent phagocytosis [41]; upregulation of *MARCO* could indicate the presence or activation of macrophages in the inflammatory infiltrates of our samples. Lastly, PERP1 is an endoplasmic reticulum resident protein, which is expressed in B lymphocytes maturing to plasma cells [42]. While lymphocytic phlebitis and mononuclear perivascular cuffs have been observed in SM and WB tissues [11,35], the majority of inflammatory cells are CD3+ T lymphocytes [2]. Future studies are required to identify the contribution of B lymphocytes to the formation of inflammatory infiltrates in both SM and WB tissues.

### 4.2. Extracellular Space and Collagen

The GO terms collagen-containing extracellular space (GO:0005615) and extracellular region (GO:0005576), in the *Cellular Component* ontology, were enriched in both the SM and WB samples. Different subunits of collagen I–III, V–VI, VIII–IX, and XII–XV were variably upregulated in SM and WB, although the magnitude of increased expression was modest and never greater than eight-fold. Indeed, our previous microscopic analysis showed increased amounts of connective tissue in the endomysium in both myopathies [10]. Together, these findings are consistent with a body of evidence implicating the quality and quantity of collagen as a contributor to the textural changes observed in breast myopathies [43,44,45]. For instance, it was suggested that the increased firmness of WB may be caused by higher concentrations of collagen (hydroxyproline) [46], higher levels of collagen cross-linking [47], or more tightly packed spatial arrangement of collagen fibers [44] when compared to normal samples. Although Baldi and colleagues reported no increase in collagen content in SM samples [9], Sanden and colleagues found that both SM and WB had an increased amount of collagen fibers compared to normal breast muscle [48]. Additionally, their study revealed that WB had thicker collagen fibers compared to SM, suggesting that collagen fibers may be more mature in WB compared to SM [48].

Supporting the results of the GO enrichment analysis, the *cell adhesion molecules* KEGG pathway was also enriched in both SM and WB groups. As part of this pathway, the cluster of differentiation 80 (*CD80*) and *CD86* genes were upregulated in both SM and WB. These molecules have a co-stimulatory function for numerous immune cells, mainly lymphocytes, and are upregulated in inflammation and autoimmune diseases [49,50]. Other upregulated genes in this enriched pathway included those of the integrin family (integrin subunit beta 2 and 7 (*ITGB2* and *7*)) and Ig superfamily (cell adhesion molecules 1 and 3 (*CADM1*, *3*); neuronal cell adhesion molecule (*NRCAM*); and vascular cell adhesion molecule (*VCAM*)). Similarly, Marchesi and colleagues documented that cell adhesion molecule 1 (CADM1) was upregulated in WS [51], while Papah and colleagues demonstrated that ITGB2 was upregulated in WB [20]. It has been suggested that cell adhesion pathways might be implicated in myofiber hypertrophy by promoting muscle cell development [52].

It remains unclear if the upregulation of cell adhesion molecules is directly implicated in the pathogenesis of broiler breast myopathies or may simply be an indicator of muscle hypertrophy, which in turn predisposes to the development of such conditions [10]. Alternatively, the upregulation of these genes may be contextual with the inflammation observed in SM and WB myopathies as cell adhesion molecules are associated with the diapedesis of white blood cells at the site of inflammation [53].

Other genes associated with the extracellular space, which were among the top 20 upregulated in both SM and WB, include C-type lectin domain family 3 (*CLEC-3A*), metalloproteinase 10 (*MMP10*), and thrombospondin-2 (*THBS2*). C-type lectins encompass a class of proteins found in the extracellular space, which carry the C-type carbohydrate recognition domains that bind proteins, lipids, or carbohydrates [54]. CLEC3A enables carbohydrate binding activity and is active in the extracellular space, and it has been shown to be involved in the ossification and development of the nucleus pulposus [55,56].

Metalloproteinases play diverse roles, including cleaving extracellular matrix components, participating in myoblast fusion and migration [57,58], and degradation of fibrous tissue [59]. *MMP10* is upregulated by thrombin in endothelial cells during inflammation [60], and it has an important role in the regeneration of striated muscle, as shown in gene knockdown experiments in mice [61]. Upregulation in myopathic samples could be caused by inflammation or regeneration or both in response to myofiber degeneration and loss [2,11,35].

THBS2 is a pro-fibrotic and anti-angiogenic matricellular protein [62], and its upregulation may be associated with the increased amounts of collagen in the endomysial space of SM and WB. It was suggested that fibrosis of WB might result from insufficient delivery of oxygen and nutrients to skeletal muscle; upregulation of *THBS2* in turn may inhibit angiogenesis, thereby leading to fibrosis in WB [2,63]. Similarly, Brothers and colleagues also reported that THBS2 was upregulated in male broilers with WB [64].

### 4.3. Tissue Morphogenesis and Proliferation

The *neuroactive ligand–receptor interaction* KEGG pathway was enriched in both SM and WB. This finding aligns with the results of Marchesi and colleagues, where they reported that the pathway was enriched in broilers affected with WB [51]. This pathway includes dopamine and serotonin receptors and is associated with signaling molecules and interaction [65]. How this pathway may affect muscle metabolism is unclear.

Cellular communication network factor 3 (*CCN3*), melan-A (*MLANA-A*), and myosin heavy chain 15 (*MYH15*) were among the 20 most upregulated genes in both SM and WB. The *CCN3* gene encodes secreted matrix-associated proteins that impair myocyte differentiation in the dermomyotome during fetal development [66], and its overexpression results in aberrant muscle repair and fibrosis [37]. Upregulation in our samples suggests altered myofiber differentiation in both SM and WB.

MLANA is located in the endoplasmic reticulum membrane and is involved in the biogenesis of melanosomes, which synthesize and store melanin pigments [67,68]. *MLANA* was upregulated in the breast muscles of black-boned chickens compared to the breast muscles of commercial broiler lines [69]; the authors of that study suggested that melanogenesis in breast muscle may result in improved flavor by production of aromatic compounds. Since our samples originated from white breast muscles, upregulation of *MLANA* was an unexpected finding.

The *MYH15* gene encodes for a protein involved in the contraction and regeneration of the avian skeletal muscle [70]. Praud and colleagues reported that MYH15 could be a potential biomarker for WB, as demonstrated by the increased expression of MYH15 in regenerating fibers within the WB-affected area [71]. This evidence suggests a potential role for *MYH15* in the pathogenesis of WB.

Taste 2 receptor (*TAS2R*) consists of a family of G-protein-coupled receptors primarily implicated in the taste of bitter compounds [72], as well as extra-gustatory effects, including thyroid function, smooth muscle relaxation, and adipogenesis [73,74,75]. While chickens appear to have a much smaller repertoire of *TAS2R* genes compared to mammals, with only three bitter receptors identified, their distribution in extra-sensory organs suggests additional functions, as observed in their mammalian counterparts [76]. The effect of *TAS2R* downregulation in the development of breast myopathies remains unclear.

Two genes involved in tissue differentiation were downregulated in both the SM and WB samples: Rho BTB containing 3 (*RHOBTB3*) and amphiphysin (*AMPH*), which are part of the Rho GTPase signaling pathway. The RHOBTB3 is a subgroup of the Rho GTPase family, which is thought to contain key regulatory molecules that link surface receptors to the organization of the actin cytoskeleton [77]. Specifically, the *RHOBTB3* encodes for a protein involved with the retrograde transport of proteins from the endosomes to the Golgi complex, as well as cell cycle regulation [78]. A similar study found that another member of the Rho GTPase family, the rho-associated coiled-coil-containing protein kinase 2 (*ROCK2*) [79], was also downregulated in WS [51]. The authors suggested that this might have affected cell adhesion and the assembly and disassembly of actin stress fibers [51,80].

Amphiphysin (*AMPH*) encodes for a protein associated with endocytosis [81,82] and membrane integrity of myocytes during muscle maturation and regeneration (including fusion of myocytes) [83,84]. Downregulation of *AMPH* may indicate the decreased regenerative ability of affected muscle, leading to repair by scarring (fibrosis).

### 4.4. Hypoxia and Oxidative Stress

Three genes associated with oxidative balance were among the 20 most highly downregulated genes in both the SM and WB samples: ChaC-glutathione-specific gamma-glutamylcyclotransferase 1 (*CHAC1*), retinol-binding protein type 1 (*RBP1*), and mitochondrial carrier protein family (solute carrier family 25, *SLC25*). The *CHAC1* genes encode a protein related to unfolded protein response and the regulation of glutathione levels and oxidative balance [85]. Brothers and colleagues identified upregulated *CHAC1* in the WB of 3-week-old male broilers and suggested that CHAC1 may degrade glutathione in the *P. major*, making the tissue more susceptible to oxidative stress, ultimately contributing to the development of myopathies [64]. The reasons for *CHAC1* downregulation in both SM and WB in our cohort are uncertain; however, differences with previous studies may reflect different stages of myopathy progression.

The product of the *RBP1* gene protects retinoids from non-specific oxidation and delivers them to specific enzymes to facilitate biosynthesis of retinoic acid [86]. *RBP1* is downregulated in humans with breast cancer as low glucose concentration and hypoxia in the tumor environment reduce its expression [87]. Downregulation of *RBP1* in our samples may reflect local hypoxia, a condition that has been associated with WB in multiple studies [8,88,89].

The *SLC25* gene family encodes for a set of proteins implicated in the exchange of molecules between the mitochondrial matrix and the cytoplasm, as well as mitochondrial viability [90]. Downregulation of this gene could negatively affect mitochondrial function and integrity, which could ultimately lead to myocyte degeneration. Ultrastructural studies of WB showed mitochondrial damage, including cristolysis and matrix dissolution [35]. Similar to our study, Marchesi and colleagues reported the downregulation of *SLC25A4* in WS [51].

### 4.5. Calcium Signaling and Phagosome

*The calcium signaling pathway* and *phagosome* KEGG pathways were enriched in WB only. Enrichment of the *calcium signaling* pathway has also been shown previously in WS [51]. An increase in cytoplasmic calcium levels could have an apoptogenic function, promoting cell death and myofiber loss, as also suggested by others [91]. The *phagosome* pathway has been shown to be enriched in denervation-induced skeletal muscle atrophy [92], and its upregulation in our samples could be associated with myofiber loss. Enrichment of this pathway could also be a sequel of inflammation, partly characterized by the influx of cells with marked phagocytic functions [81].

## 5. Conclusions

Our findings show that both SM and WB presented with similar transcriptomic profiles. While several GO terms and KEGG pathways were enriched, there was substantial overlap with genes variably associated with inflammation/immune response and the extracellular space (the same genes can be part of multiple enriched terms and pathways). This suggests that inflammation and fibrosis were the primary drivers of the transcriptomics changes observed in our samples. As both inflammation and fibrosis are the stereotypical end result of many different types of tissue injury [37], it is unclear if similar transcriptomic profiles between SM and WB indicate similar disease mechanisms, or a common outcome. Future studies to correlate transcriptomic results with the severity of histological changes may be useful. While the lack of histological analysis may be a limitation of the present study, in our experience, microscopic differentiation of breast myopathies presents considerable challenges [10] and was not adopted. Instead, macroscopic classification was carried out as it was considered more relevant to field presentation. Another limitation of this study is the absence of clear clustering of WB samples in the PCA plots and heatmaps, even when increasing the FDR threshold. Because of this dispersion, our approach might not have identified DEGs between the SM and WB groups. It is unclear why the WB cohort presented a higher variance. The more subjective nature of the WB scoring (compared to the clearer visual determination of spaghetti meat) might have played a role. It should also be kept in mind that WB in Canadian broilers does not usually present with the degree of severity observed in other countries, such as the US, where birds can be slaughtered at higher weights [7].

Our transcriptomic results suggest that the two myopathies share at least in part a common pathogenesis. It is even possible that WB may predispose to the development of SM: despite muscle fibrosis, WB samples could suffer from a weakened extracellular scaffold, leading to myofiber unravelling when physical stresses are applied to the fillets at the processing plant, which occurs during defeathering, deboning, and tumbling during chilling. This interpretation is consistent with the macroscopic features of SM being clearly a post-mortem change (i.e., lack of hemorrhage and severe myonecrosis despite tissue loss and separation). A study from our group showed that water chilling was positively correlated with the frequency of SM [3], further supporting the relationship with a processing method.

In summary, SM and WB share common transcriptomic profiles. Additional studies are needed to understand the contribution of the protein products of DEG to the pathogenesis, and to understand if these macroscopically distinct myopathies represent separate disease processes or different phenotypes of the same condition, exacerbated by postmortem manipulation.

## Figures and Tables

**Table 1:** List of the top 20 upregulated (top of the list) and downregulated (bottom of the list) genes, with associated fold changes, in samples affected by spaghetti meat (SM) compared to Normal samples. The false discovery rate was used to control for multiple comparisons, with an adjusted *p*-value threshold of 0.1.

Symbol	Description	Log2 Fold Change	Adjusted *p*-Value
*PTX3*	Pentraxin 3	4.91	2.69^−04
*MLANA*	Melan-A	4.72	3.10^−03
*CLEC3A*	C-type lectin domain family 3 member A	4.67	3.18^−03
*GABRA5*	Gamma-aminobutyric acid type A receptor alpha5 subunit	4.58	1.35^−04
*IL5RA*	Interleukin 5 receptor subunit alpha	4.51	2.73^−05
*CCN3*	Cellular Communication Network Factor 3	4.43	2.12^−02
*ASB18*	Ankyrin repeat and SOCS box containing 18	4.39	7.61^−02
*MARCO*	Macrophage receptor with collagenous structure	4.13	6.11^−04
*PERP1*	PERP1, TP53 apoptosis effector	3.95	9.48^−04
*KRT24*	Keratin 24	3.93	1.83^−03
*PGR2/3*	Tandem PRG2/PRG3 gene pair	3.89	1.50^−04
*THBS2*	Thrombospondin 2	3.87	2.32^−02
*GPR141*	G protein-coupled receptor 141	3.77	3.07^−05
*DSG2*	Desmoglein 2	3.75	7.67^−03
*MDK*	Midkine (neurite growth-promoting factor 2)	3.70	8.31^−04
*RSPO4*	R-spondin 4	3.40	6.08^−04
*MMP10*	Matrix metallopeptidase 10	3.33	1.07^−02
*CYP1C1*	Cytochrome P450 family 1 subfamily C polypeptide 1	3.31	2.16^−03
*MYH15*	Myosin, heavy chain 15	3.28	7.79^−03
*CTHRC1*	Collagen triple helix repeat containing 1	3.23	1.60^−02
*AMPH*	Amphiphysin	−3.25	6.88^−04
*CHAC1*	ChaC glutathione specific gamma-glutamylcyclotransferase 1	−2.37	9.63^−03
*PGPEP1L*	Pyroglutamyl-peptidase I like	−2.37	4.93^−03
*SLC25A30*	Solute carrier family 25 member 30	−2.19	2.16^−03
*7SK*	7SK RNA	−2.08	2.81^−03
*FGF1*	Fibroblast growth factor 1	−1.90	2.78^−04
*GPR160*	G protein-coupled receptor 160	−1.89	1.07^−02
*FRMD5*	FERM domain containing 5	−1.89	2.57^−02
*AMBP*	Alpha-1-microglobulin/bikunin precursor	−1.86	5.11^−03
*ESR2*	Estrogen receptor 2	−1.83	6.96^−03
*HIBADH*	3-hydroxyisobutyrate dehydrogenase	−1.82	7.91^−05
*TAS2R7*	Taste receptor, type 2, member 7	−1.80	5.01^−02
*TENT5B*	Terminal nucleotidyltransferase 5B	−1.78	7.22^−04
*GLUL*	Glutamate-ammonia ligase	−1.75	9.58^−04
*KCTD20*	Potassium channel tetramerization domain containing 20	−1.75	6.25^−04
*RHOBTB3*	Rho related BTB domain containing 3	−1.73	5.99^−03
*RBP1*	Retinol binding protein 1	−1.73	3.96^−03
*FSTL4*	Follistatin like 4	−1.72	8.36^−02
*GJB6*	Gap junction protein beta 6	−1.61	7.24^−02
*ARRDC2*	Arrestin domain containing 2	−1.61	7.49^−03

**Table 2:** List of the top 20 upregulated (top of the list) and downregulated (bottom of the list) genes, with associated fold changes, in samples affected by woody breast (WB) compared to Normal samples. The false discovery rate was used to control for multiple comparisons, with an adjusted *p*-value threshold of 0.1.

Symbol	Description	Log2 Fold Change	Adjusted *p*-Value
*ASB18*	Ankyrin repeat and SOCS box containing 18	5.21	6.21^−02
*MYL3*	Myosin, light chain 3, alkali; ventricular, skeletal, slow	4.56	6.53^−02
*CCN3*	Cellular Communication Network Factor 3	3.77	8.66^−02
*PTX3*	Pentraxin 3	3.66	1.63^−02
*CLEC3A*	C-type lectin domain family 3 member A	3.62	5.00^−02
*MLANA*	Melan-A	3.58	5.54^−02
*IL5RA*	Interleukin 5 receptor subunit alpha	3.54	1.32^−02
*MARCO*	Macrophage receptor with collagenous structure	3.39	1.65^−02
*THBS2*	Thrombospondin 2	3.32	8.92^−02
*KRT24*	Keratin 24	3.25	2.78^−02
*GABRA5*	Gamma-aminobutyric acid type A receptor alpha5 subunit	3.24	1.83^−02
*CSRP3*	Cysteine and glycine rich protein 3	3.20	1.96^−02
*PERP1*	PERP1, TP53 apoptosis effector	3.20	2.31^−02
*DSG2*	Desmoglein 2	3.12	5.38^−02
*CALB2*	Calbindin 2	3.12	6.36^−02
*MYH15*	Myosin, heavy chain 15	3.12	3.05^−02
*MYL10*	Myosin, light chain 10, regulatory	3.12	4.74^−02
*MMP7*	Matrix metallopeptidase 7	3.11	3.74^−02
*IL8L1*	Interleukin 8-like 1	3.05	4.00^−02
*MMP10*	Matrix metallopeptidase 10	3.00	4.79^−02
*TAS2R7*	Taste receptor, type 2, member 7	−3.20	1.99^−02
*AMPH*	Amphiphysin	−2.62	1.44^−02
*FRMD5*	FERM domain containing 5	−2.39	2.38^−02
*GPR160*	G protein-coupled receptor 160	−2.12	2.30^−02
*CHAC1*	ChaC glutathione specific gamma-glutamylcyclotransfease 1	−2.08	4.17^−02
*FSTL4*	Follistatin like 4	−2.07	7.75^−02
*RBP1*	Retinol binding protein 1	−2.05	1.32^−02
*KY*	Kyphoscoliosis peptidase	−2.05	7.76^−02
*RHOBTB3*	Rho related BTB domain containing 3	−1.97	1.65^−02
*PGPEP1L*	Pyroglutamyl-peptidase I like	−1.72	4.62^−02
*VAMP1*	Vesicle-associated membrane protein 1 (synaptobrevin 1)	−1.70	1.70^−02
*FGF1*	Fibroblast growth factor 1	−1.70	1.32^−02
*GADL1*	Glutamate decarboxylase like 1	−1.69	7.07^−02
*gga-mir-1754*	Gga-mir-1754	−1.67	1.55^−02
*USH2A*	Usherin	−1.63	4.23^−02
*SBK2*	SH3 domain binding kinase family member 2	−1.60	2.06^−02
*NRGN*	Neurogranin (protein kinase C substrate, RC3)	−1.59	3.13^−02
*THSD7B*	Thrombospondin type 1 domain containing 7B	−1.56	2.94^−02
*NMNAT3*	Nicotinamide nucleotide adenylyltransferase 3	−1.53	2.41^−02
*SLC25A30*	Solute carrier family 25 member 30	−1.52	4.71^−02

**Table 3:** Highly differentially regulated genes in common between spaghetti meat (SM) and woody breast (WB) samples compared to Normal samples. Up- or downregulated genes are at the top and bottom of the list, respectively. The false discovery rate was used to control for multiple comparisons, with an adjusted *p*-value threshold of 0.1.

Symbol	Description	SM vs. Normal		WB vs. Normal	
		Log2 Fold Change	Adjusted *p*-Value	Log2 Fold Change	Adjusted *p*-Value
*PTX3*	Pentraxin 3	4.91	2.69^−04	3.66	1.63^−02
*MLANA*	Melan-A	4.72	3.10^−03	3.58	5.54^−02
*CLEC3A*	C-type lectin domain family 3 member A	4.67	3.18^−03	3.62	5.00^−02
*GABRA5*	Gamma-aminobutyric acid type A receptor alpha5 subunit	4.58	1.35^−04	3.24	1.83^−02
*IL5RA*	Interleukin 5 receptor subunit alpha	4.51	2.73^−05	3.54	1.32^−02
*CCN3*	Cellular Communication Network Factor 3	4.43	2.12^−02	3.77	8.66^−02
*ASB18*	Ankyrin repeat and SOCS box containing 18	4.39	7.61^−02	5.21	6.21^−02
*MARCO*	Macrophage receptor with collagenous structure	4.13	6.11^−04	3.39	1.65^−02
*PERP1*	P53 apoptosis effector related to PMP-22	3.95	9.48^−04	3.20	2.31^−02
*KRT24*	Keratin 24	3.93	1.83^−03	3.25	2.78^−02
*THBS2*	Thrombospondin 2	3.87	2.32^−02	3.32	8.92^−02
*DSG2*	Desmoglein 2	3.75	7.67^−03	3.12	5.38^−02
*MMP10*	Matrix metallopeptidase 10	3.33	1.07^−02	3.00	4.79^−02
*MYH15*	Myosin, heavy chain 15	3.28	7.79^−03	3.12	3.05^−02
*FSTL4*	Follistatin like 4	−1.72	8.36^−02	−2.07	7.75^−02
*RBP1*	Retinol binding protein 1	−1.73	3.96^−03	−2.05	1.32^−02
*RHOBTB3*	Rho related BTB domain containing 3	−1.73	5.99^−03	−1.97	1.65^−02
*TAS2R7*	Taste receptor, type 2, member 7	−1.80	5.01^−02	−3.20	1.99^−02
*FRMD5*	FERM domain containing 5	−1.89	2.57^−02	−2.39	2.38^−02
*GPR160*	G protein-coupled receptor 160	−1.89	1.07^−02	−2.12	2.30^−02
*FGF1*	Fibroblast growth factor 1	−1.90	2.78^−04	−1.70	1.32^−02
*SLC25A30*	Solute carrier family 25 member 30	−2.19	2.16^−03	−1.52	4.71^−02
*CHAC1*	ChaC glutathione specific gamma-glutamylcyclotransferase 1	−2.37	9.63^−03	−2.08	4.17^−02
*PGPEP1L*	Pyroglutamyl-peptidase I like	−2.37	4.93^−03	−1.72	4.62^−02
*AMPH*	Amphiphysin	−3.25	6.88^−04	−2.62	4.62^−02

**Table 4:** Expression of different collagen genes in both spaghetti meat (SM) and woody breast (WB) samples compared to Normal samples. The false discovery rate was used to control for multiple comparisons, with an adjusted *p*-value threshold of 0.1.

Symbol	Description	SM vs. Normal		WB vs. Normal	
		Log2 Fold Change	Adjusted *p*-Value	Log2 Fold Change	Adjusted *p*-Value
Collagen genes upregulated in both SM and WB					
*COL1A1*	Collagen type I alpha 1 chain	1.40	1.14^−02	1.35	3.40^−02
*COL1A2*	Collagen type I alpha 2 chain	1.36	1.21^−02	1.22	4.47^−02
*COL22A1*	Collagen type XXII alpha 1 chain	1.23	4.14^−03	1.07	3.13^−02
*COL3A1*	Collagen type III alpha 1 chain	1.36	6.70^−03	1.00	6.14^−02
*COL5A2*	Collagen type V alpha 2 chain	1.04	8.30^−03	0.81	5.91^−02
*COL6A1*	Collagen type VI alpha 1 chain	1.21	2.74^−03	0.97	3.06^−02
*COL6A2*	Collagen type VI alpha 2 chain	1.14	2.36^−03	0.93	2.70^−02
*COL6A3*	Collagen type VI alpha 3 chain	1.33	7.15^−04	0.89	3.10^−02
*COL8A1*	Collagen type VIII alpha 1 chain	1.41	3.10^−03	1.13	3.52^−02
*COL8A2*	Collagen type VIII alpha 2 chain	1.67	2.58^−02	1.64	5.87^−02
*COL9A2*	Collagen type IX alpha 2 chain	1.43	1.45^−03	1.18	2.46^−02
*COL12A1*	Collagen type XII alpha 1 chain	2.12	1.50^−02	1.91	5.26^−02
*COL13A1*	Collagen type XIII alpha 1 chain	1.34	1.39^−03	0.91	4.84^−02
Collagen genes upregulated in SM but not in WB					
*COL14A1*	Collagen type XIV alpha 1 chain	1.17	6.57^−03	NA	NA
*COL5A1*	Collagen type V alpha 1 chain	0.83	2.84^−02	NA	NA

## Data Availability

All FASTQC files of RNA sequencing data are available from the University of Guelph Research Data Repositories (accession number, https://doi.org/10.5683/SP3/LDBRRW).

## Supplementary Materials

The following supporting information can be downloaded at https://www.mdpi.com/article/10.3390/ani14020176/s1, Table S1: Summary of RNA sequencing, quality control, and alignment results; Table S2: List of upregulated (bold) and downregulated (regular) genes included in each significantly enriched Kyoto Encyclopedia of Genes and Genomes (KEGG) pathway in spaghetti meat (SM) and woody breast (WB) compared to Normal; Figure S1: Classification of samples: (a) fillet with spaghetti meat; (b) fillet with woody breast; (c) normal fillet.
