# Supplementary material for: Transcriptomic Profiles of Pectoralis major Muscles Affected by Spaghetti Meat and Woody Breast in Broiler Chickens
Source: Animals (Basel). 2024 Jan 5;14(2):176. doi: 10.3390/ani14020176 (PMC10812457; doi:10.3390/ani14020176)
Supplement: Supplementary file 1 [file animals-14-00176-s001.zip › animals-2793907-supplementary.pdf]

Table S1. Summary of RNA sequencing, quality control and alignment results.

Sample	Number of raw reads	Number of surviving reads	Surviving reads (%)	Number of mapped reads	Mapped reads (%)	Genes
1	213,401,856	213,255,838	99.93	184,777,470	86.65	11,187
2	194,164,712	193,983,724	99.91	168,511,777	86.87	11,243
3	205,093,538	204,895,900	99.90	177,443,813	86.60	10,960
4	198,327,514	198,158,896	99.92	173,438,061	87.52	11,188
5	216,353,356	216,161,442	99.91	191,537,838	88.61	11,157
6	195,625,952	195,477,176	99.92	167,666,370	85.77	11,151
7	178,343,004	178,239,598	99.94	154,740,375	86.82	11,375
8	204,412,592	204,316,902	99.95	177,594,272	86.92	11,188
9	181,709,148	181,538,710	99.91	154,872,490	85.31	10,943
10	218,761,844	218,622,872	99.94	191,540,034	87.61	11,305
11	237,510,502	237,343,172	99.93	215,292,991	90.71	11,746
12	175,574,102	175,463,934	99.94	155,722,734	88.75	11,536
13	185,868,090	185,724,908	99.92	164,628,708	88.64	11,307
14	173,424,146	173,324,374	99.94	158,098,991	91.22	11,746
15	220,764,806	220,628,522	99.94	191,482,255	86.79	11,219
16	174,177,176	174,080,220	99.94	153,658,876	88.27	11,303
17	213,875,066	213,675,818	99.91	182,897,846	85.60	11,231
18	191,855,660	191,738,392	99.94	165,977,330	86.56	11,129
19	205,782,596	205,656,296	99.94	178,054,232	86.58	11,456
20	235,088,342	234,909,086	99.92	202,033,036	86.00	11,433
21	198,469,448	198,327,096	99.93	173,854,847	87.66	11,463
22	189,305,288	189,184,896	99.94	164,478,279	86.94	11,423
23	193,761,902	193,628,786	99.93	167,514,977	86.51	11,303
24	161,705,438	161,609,070	99.94	140,385,186	86.87	11,439
25	188,780,844	188,649,956	99.93	169,516,537	89.86	11,650
26	218,848,164	218,717,794	99.94	194,601,491	88.97	11,649
27	215,266,218	215,137,846	99.94	193,424,579	89.91	11,650
28	246,025,724	245,898,274	99.95	224,652,618	91.36	11,766
29	178,457,600	178,322,804	99.92	158,799,601	89.05	11,535
30	175,353,224	175,222,624	99.93	156,403,655	89.26	11,490

Table S2. List of upregulated (bold) and downregulated (regular) genes included in each significantly enriched Kyoto Encyclopedia of Genes and Genomes (KEGG) pathway, in spaghetti meat (SM) and woody breast (WB) compared to Normal.

KEGG pathway	DEGs in SM vs. Normal comparison	DEGs in WB vs. Normal comparison
Cytokine-cytokine receptor interaction	**BMP2, CCL17, CCL19, CCL20, CCL4, CCL5, CCR2, CCR4, CCR5, CCR6, CCR7, CCR8, CD40, CD40LG, CLCF1, CSF1R, CSF2RA, CSF3R, CX3CR1, CXCL12, CXCL14, CXCR1, CXCR4, FAS, FLT3,** GHR, **HGF, IFNAR1, IL11, IL12RB2, IL11, IL12RB2, IL13RA1, LI17RA, IL18, IL18R1, IL18RAP, IL1B, IL1R1, IL1R2, IL1RAP, IL20RA, IL21R, IL22RA2, IL2RA, IL2RB, IL2RG, IL5RA, IL7R, INHBC,** LIFR, **PDGFRA, PDGFRB, RELT, TGFB1, TGFB3, TGFBR2, TNFRSF11A, TNFRSF13B, TNFRSF18, TNFRSF19, TNFRSF1A, TNFRSF1B, TNFSF8, XCL1, XCR1**	**CCL17, CCL19, CCL4, CCL5, CCR4, CCR5, CCR6, CCR7, CCR8, CD40LG, CLCF1,** CNTFR, **FLT3,** GHR, **IL10RA, IL11, IL13RA1, IL17RA, IL18, IL18R1, IL1B, IL1R2, IL1RAP, IL20RA, IL21R, IL22RA2, IL2RA, IL2RB, IL2RG, IL5RA, IL7R, TGFB1, TGFBR2, TNFRSF18, TNFRSF19, TNFSF13B, TNFSF15, TNFSF8, XCL1, XCR1**
Calcium signaling pathway		**ADCY2, ADCY7, ATP2A2, CACNA1C, CACNA1E, F2R, HTR2A, HTR2B, ITPR2, MYLK,** NOS1, **P2RX1, P2RX2, P2RX3, P2RX4, P2RX7,** PDE1B, **PDGFRA,** PHKA1, **PHKA2,** PHKB, PHKG1, PLCB1, **PLCB2, PLCG2, PLN,** PPP3CA, PPP3CC, **PTAFR, PTGFR,** SLC25A4, **SLC8A1, SPHK1,** TNNC1, TNNC2, VDAC1
Neuroactive ligand-receptor interaction	**ADORA1, ADORA3, ADRA2A, ADRA2C,** ADRB2, **AGTR2, C3AR1, CHRM4, CHRNA1,** CHRNA4, CHRNA5, **CHRND, CHRNG, CNR1, EDNRA, F2R, F2RL1, FSHR, GABRA1, GABRA5,** GHR, **GPR156, GPR50, GZMA, HRH2, HTR2A, HTR2B, HTR6, HTR7, LHCGR, LPAR1, LPAR3,** LPAR6, **LTB4R, P2RX1, P2RX2, P2RX3, P2RX4, P2RX7,** P2RY1, **P2RY13, P2RY4, P2RY8, PTAFR, PTGDR,** PTGER3, **PTGER4, RXFP1, S1PR3, S1PR4, SSTR2, TSPO, VIPR1**	**ADORA1,** ADRB2, **C3AR1, CHRM4, CHRNA1,** CHRNA4, CHRNA5, **CNR1,** CRHR2, **GABRA1, GABRA5,** GHR, **LTB4R, PTGDR, PTGER4, PTGFR, S1PR3, S1PR4**
**KEGG pathway**	**DEGs in SM vs. Normal comparison**	**DEGs in WB VS. Normal comparison**
Phagosome		**ANXA1, APPL2, ARHGAP25, BIN2, BTK, C3, CAMK1D, CNN2, COLEC12, CORO1C, CRP, DOCK2, F2RL1, FCER1G, GAS6, GSN, HAVCR1, HCK, IL1B, IL2RB, IL2RG, IRF8, ITGAV, ITGB2, ITGB3, LDLR, LIMK1, LPR1,** LYAR, **LYN, MARCO, MERTK, MYO1G, NCF2, NCF4, NCKAP1L,** NR1H3, **P2RX7, P2RY6, PLCG2, PLD4, PTPRC, PTPRJ, PTX3, RAB31, RAB39A, RAB7B, RAC2, ROHG, SIRPA,** SOD1, **SRC, SYK, SYT11, SYT7, THBS1, TIMD4, TLR4, TREM2, TUB, VAV2, VAV3**
Oxidative phosphorylation	ATP5MC1, ATP5PB, ATP5PD, ATP5PF, ATP6V0A1, **ATP6V0D2,** COX10, COX5B, COX6C, COX7A2L, COX7C, COX10, LHPP, NDUFA1, NDUFA2, NDUFA4, NDUFA8, NDUFB4, NDUFB5, NDUFC2, NDUFS1, NDUFS3,	

	NDUFS4, NDUFS6, NDUFS7, NDUFS8, SDHA, SDHB, SDHC, **TCIRG1**, UQCR11, UQCRB, UQCRC2, UQCRFS1, UQCRHL, UQCRQ	
Cell adhesion molecules	**CD28, CD40LG, CD80, CD86, CD8A, ITGB2, ITGB7**	**CADM1, CADM3, CD4, CD40LG, CD80, CD86, CD8A, CTLA4, ITGB2, ITGB7, NRCAM, VCAN**
Intestinal immune network for IgA production	**CD28, CD40, CD40LG, CD80, CD86, CXCL12, CXCR4, ICOS, ICOSLG, ITGA4, TGFB1, TNFRSF13B, TNFSF13B**	

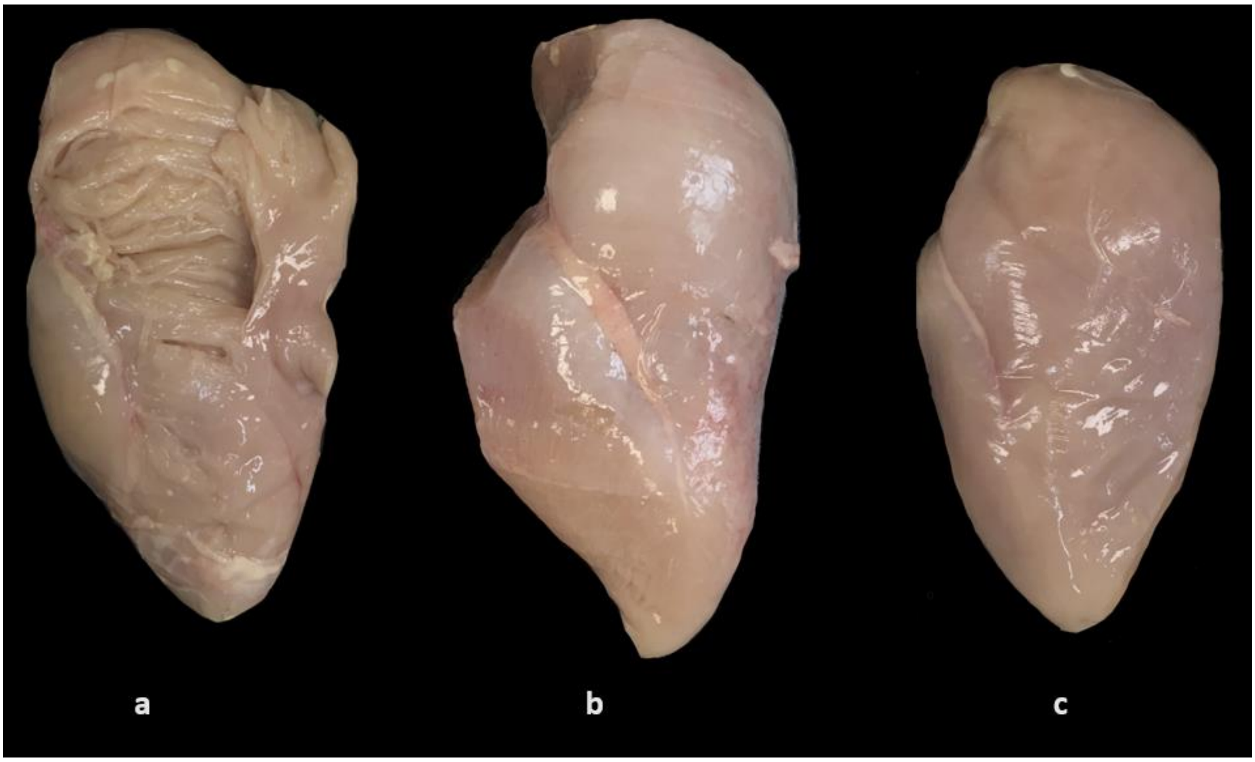

Figure S1. Classification of samples: (**a**) fillet with spaghetti meat; (**b**) fillet with woody breast; (**c**) normal fillet.
